# Predicting the risk of depression among adolescents in Nepal using a model developed in Brazil: the IDEA Project

**DOI:** 10.1007/s00787-020-01505-8

**Published:** 2020-03-12

**Authors:** Rachel Brathwaite, Thiago Botter-Maio Rocha, Christian Kieling, Kamal Gautam, Suraj Koirala, Valeria Mondelli, Brandon Kohrt, Helen L. Fisher

**Affiliations:** 1 Social Genetic and Developmental Psychiatry Centre, Institute of Psychiatry, Psychology and Neuroscience, King’s College London, 16 De Crespigny Park, London, SE5 8AF UK; 2 Department of Psychiatry, Universidade Federal Do Rio Grande Do Sul, Porto Alegre, Brazil; 3 Child and Adolescent Psychiatry Division, Hospital de Clínicas de Porto Alegre, Porto Alegre, Brazil; 4 Transcultural Psychosocial Organization Nepal (TPO Nepal), Kathmandu, Nepal; 5 Department of Psychological Medicine, Institute of Psychiatry, Psychology and Neuroscience, King’s College London, London, UK; 6 Division of Global Mental Health, George Washington University, Washington, DC USA

**Keywords:** Adolescence, External validation, LMIC, Mental health, Prediction model, Risk calculator

## Abstract

The burden of adolescent depression is high in low- and middle-income countries (LMICs), yet research into prevention is lacking. Development and validation of models to predict individualized risk of depression among adolescents in LMICs is rare but crucial to ensure appropriate targeting of preventive interventions. We assessed the ability of a model developed in Brazil, a middle-income country, to predict depression in an existing culturally different adolescent cohort from Nepal, a low-income country with a large youth population with high rates of depression. Data were utilized from the longitudinal study of 258 former child soldiers matched with 258 war-affected civilian adolescents in Nepal. Prediction modelling techniques were employed to predict individualized risk of depression at age 18 or older in the Nepali cohort using a penalized logistic regression model. Following a priori exclusions for prior depression and age, 55 child soldiers and 71 war-affected civilians were included in the final analysis. The model was well calibrated, had good overall performance, and achieved good discrimination between depressed and non-depressed individuals with an area under the curve (AUC) of 0.73 (bootstrap-corrected 95% confidence interval 0.62–0.83). The Brazilian model comprising seven matching sociodemographic predictors, was able to stratify individualized risk of depression in a Nepali adolescent cohort. Further testing of the model’s performance in larger socio-culturally diverse samples in other geographical regions should be attempted to test the model’s wider generalizability.

**Electronic supplementary material:**

The online version of this article (10.1007/s00787-020-01505-8) contains supplementary material, which is available to authorized users.

## Introduction

Major depressive disorder (MDD) is a leading underlying cause of disability worldwide [1]. MDD increases the future risk of developing chronic diseases including diabetes mellitus, cardiovascular disease, and stroke [2], and is a major contributor to death by suicide globally [1]. MDD commonly emerges during adolescence [3, 4], yet despite receiving treatment, many adolescents experience a relapse of depression [5]. Consequently, functional impairment and increased risk of suicide continue into adulthood [6]. Given that by age 18, lifetime prevalence of MDD is approximately 11% [7, 8], this reinforces the need for research to prevent the onset of depression in the adolescent period to facilitate early intervention and avoid long-term health and socioeconomic disadvantage [9]. Therefore, it is important to ascertain which adolescents are most at risk of developing depression to effectively target interventions to prevent its onset [10].

Factors such as family psychiatric history [11], childhood maltreatment [12], female gender [13], chronic pain or illnesses [14], are associated with an increased risk of depression among adolescents. However, these risk factors have mainly been considered in isolation and knowledge about the combination of factors that best predicts the onset of depression in this critical developmental period is limited. One method for estimating combined risk of future events is prediction modelling. Advantages of this method over standard regression approaches include prediction of new or future risks of an outcome at the individual rather than group level, while accounting for a wide combination of predictors simultaneously [15]. Examples of widely used prediction models include: the Framingham Risk Score which is utilized in primary care settings to predict individualized future risk of developing a first cardiovascular event in 10 years among individuals free of cardiovascular disease [16], and the Gail model used to predict 5-year and lifetime risk of invasive breast cancer in healthy women [17]. The development of models to predict individualized risk of future psychiatric health outcomes is expanding (though mainly limited to predicting psychosis), but is still in its infancy for depression and other mental illnesses [18, 19].

Models that predict MDD have conventionally been derived in adult not adolescent populations [20–23], and limited to patients who have experienced chronic or life-threatening medical conditions [24–27] or to predict recurrence of depression [28, 29]. The Chicago Adolescent Risk Assessment is one known model developed in a US adolescent population to predict 1-year risk of depression in adolescents [30]. However, this model was not externally validated and overall few researchers have externally validated depression prediction models in relatively similar but socio-culturally or geographically diverse populations to test the model’s predictive accuracy [21, 22].

Our group has recently developed a multivariable prediction model to predict individualized risk of developing depression in late adolescence using data from the population-based 1993 Pelotas Birth Cohort in south Brazil (Pelotas model) [31]. Although the Pelotas model performed well in predicting depression in the cohort from Brazil, a middle-income country, its ability to accurately predict depression among adolescents in resource poor, low-income settings is unknown. Nepal presents an opportunity to test this because it is considered to be one of the least developed countries globally, with ~ 15% of the population below the income poverty line [32]. Additionally, Nepal has suffered an 11-year (1996–2006) Civil War (also known as the “People’s War”) between the Communist Party of Nepal (Maoist) and the government of Nepal, resulting in the killings of over 17,000 people [33]. Nepal comprises a large youth population (> 50% of the population younger than age 25) transitioning into adulthood [34]. During the war, several thousand children were drafted by the Maoist People’s Army to be soldiers, sentries, spies, cooks, porters, and messengers [35, 36]. Due to the negative impact to health, social well-being, and financial stability imposed during the war, one consequence has been a high prevalence of adolescent depression observed in Nepal [37], leading to poor quality of life [37], and high levels of suicidal ideation [38]. However, not all adolescents exposed to this environment developed depression, and we do not know which Nepali adolescents are at higher risk of developing depression in the future.

Therefore, we assessed the Pelotas model’s ability to predict depression in late adolescence in an existing adolescent cohort from Nepal, to evaluate the performance of the model in a socio-culturally different lower income setting.

## Methods

### Description of study setting and recruitment of study cohort

We used quantitative data from a longitudinal study of child soldiers and matched civilians in Nepal [38–41]. The study was conducted by Transcultural Psychosocial Organization Nepal (TPO Nepal). At the end of the war, some former child soldiers who returned home participated in reintegration programs sponsored by UNICEF in eight districts (Dhankuta, Sindhuli, Makwanpur, Chitwan, Rupandehi, Kapilbastu, Dhading, and Dolakha) of Nepal [40]. The programs included formal education, vocational skill training, apprenticeships, or business development skill training to enhance their income-generating abilities [42]. This project identified and recruited a Nepali cohort of former child soldiers, using lists of names of child soldiers provided by UNICEF-associated human rights organizations.

Former child soldiers were recruited into the study if they were younger than 18 years old at study enrolment, served as a soldier for at least 1 month during the war, and if consent was granted by their caregiver and oral assent by the child. The first 30 names of former child soldiers enrolled in reintegration programs from each of the eight districts were invited to participate in the study. A cohort of war-affected civilian children, matched on age, sex, ethnicity, and educational level, who were not associated with armed forces and groups (civilian children) were recruited from school records. Civilian status was confirmed via interviews and name checking on the child soldiers’ lists. None of the participants had previously received psychosocial support before joining the study.

The Nepali adolescent cohort was aged 11–18 years at baseline (wave 1) in 2007 (*N* = 516), comprising 258 former child soldiers who returned home from the war, matched with 258 war-affected civilian adolescents [40]. This cohort was followed up 1 year later in 2008 (wave 2: *N* = 456), and 5 years later in 2012 (wave 3: *N* = 290) [39]. Due to the high illiteracy rate among the study population, trained researchers administered questionnaires to former child soldiers and civilian children to collect data on a range of characteristics during the child soldiers’ longitudinal research.

### Outcome assessment

The outcome in our analysis was of the presence of clinically relevant depression at age 18 or older. Depression was assessed using the Nepali version of the Depression Self Rating Scale (DSRS) for children at all time points [43, 44]. This tool utilized self-reported ratings of ‘Mostly’, ‘Sometimes’, or ‘Never’ for 18 items used to measure depression symptoms in the past week. Scores can range from 0 to 36, with a cut-off score of 14 and above considered as indicating clinical depression in the Nepali population [area under the curve (AUC) = 0.82, sensitivity = 0.71, specificity = 0.81] [44].

### Data harmonization

For this analysis, an a priori decision was made to select predictors that most closely matched those in the existing prediction model derived in the 1993 Pelotas birth cohort in Brazil [31, 45]. The 11 predictors in the Pelotas model were ‘biological sex’, ‘skin colour’, ‘drug use’, ‘school failure’, ‘social isolation’, ‘fight involvement’, ‘relationship with mother’, ‘relationship with father’, ‘relationship between parents’, ‘childhood maltreatment’, and ‘ran away from home’. For the ‘skin colour’ Pelotas variable, we used caste/ethnicity in Nepal, with low caste (*Dalit*) considered the at-risk group in comparison to high caste (*Brahman/Chhetri*) and ethnic minority (*Janajati*) groups. This categorization was based on identification of low caste as a risk factor in multiple prior studies in Nepal [37, 46]. More details on the availability of matching predictors and how they were assessed in the Pelotas and Nepali samples are provided in Table S1.

### Selection of Nepali sample to be included in prediction modelling analysis

Because the Nepali cohort was not a birth cohort and adolescents were different ages (11–18 years) at baseline (wave 1), we assessed exposure to potential risk factors if they occurred before age 18 and evaluated the outcome of depression at wave 3 when participants were aged 18 or older. Adolescents were, therefore, included in the analysis if they met the following criteria: younger than 18 at wave 1 or 2; did not have evidence of depression at wave 1 or 2; aged 18 or older at wave 3; and assessed for depression at all waves. Adolescents were excluded if they were: lost-to-follow-up at waves 2 or 3; older than 18 at wave 1 or 2; younger than 18 at wave 3; or had evidence of depression at waves 1 or 2 (see Fig. S1 for a flowchart explaining the selection of the final sample included in the analysis). The final sample included in the analysis comprised 126 adolescents (71 civilians and 55 former child soldiers).

### Data analysis

Data management was performed using STATA, version 15.1 [47]. All models were implemented using the R Statistics software, version 3.5.3 [48]. The data analysis comprised several steps. First, the linear predictor from the penalized logistic regression model [which used penalized maximum likelihood estimation (PMLE)], developed in the Pelotas cohort (Pelotas model) was recalculated using only the same predictor variables that were also available in the Nepali dataset. This model was then applied in the Nepali dataset to assess the adequacy of its performance (standard external validation). Second, due to differences in the prevalence of depression in late adolescence between the Pelotas and Nepali cohorts, the model intercept became mis-calibrated. The Pelotas model intercept (baseline risk) was, therefore, adjusted through recalibration so that the average predicted probability was equal to the observed frequency of depression in the Nepali cohort (adjusted external validation) [49]. Third, to account for different strengths of predictors between the Pelotas and the Nepali cohorts, the regression coefficients for the predictors were re-estimated in the Nepali dataset instead of the Pelotas dataset and a new refitted linear predictor was obtained (refitted model). This represents the performance of the model if the regression coefficients from the Pelotas cohort were the same as the regression coefficients in the Nepali cohort [50].

#### Sensitivity analyses

As a sensitivity analysis, we explored whether the Pelotas model’s ability to predict depression differed according to child soldier status, given that being a child soldier increased the risk of depression among Nepali youth [37]. To do so, we fitted a logistic regression model which comprised the linear predictor derived from the Pelotas model, the child soldier variable, and their interaction term.

Net reclassification improvement (NRI) methods were also used to assess improvement in the model performance by assessing to what extent adolescents were correctly reclassified into high- and low-risk depression categories by the inclusion of child soldier status in the Pelotas model [51].

### Evaluation of model performance

The predictive performance of the Pelotas prediction model externally validated in the Nepali dataset was evaluated by assessing: (1) calibration—the agreement between observed depression in the Nepali dataset and predicted probability of depression from the Pelotas model; and (2) discrimination—how well the prediction model can differentiate those with depression from those without depression [52]. Model calibration was evaluated visually via calibration plots. We referred to the values of (1) calibration-in-the-large—comparison of the average of all predicted probabilities with the average observed depression cases in the Nepali dataset, with values closer to zero indicating better model performance; and (2) calibration slope—measure of agreement between observed depression and predicted risk of depression for all predictors in the Nepali dataset (a perfect model has a calibration slope of 1; [53]). A Chi-square test to measure unreliability of the calibration accuracy was performed to assess whether there was a statistically significant difference between the model predictions and the 45° line [53]. We assessed discrimination using the receiver operator characteristic (ROC) curve. An area under the curve (AUC) value of 0.5 indicates that a model does not discriminate better than chance, while 1 indicates that a model discriminates perfectly. Guidelines suggest AUC values over 0.7 represent a good model whereas values ≥ 0.8 indicate strong models [54]. Overall, model performance was assessed using the Brier score [52]. This calculates the average squared difference between the predicted probability of depression and the actual probability of depression [55]. A Brier score of 0% represents a perfect model.

Note, unlike traditional regression models, penalized regression models do not permit interpretation of coefficients for the individual predictors included in the model. This is because applying a penalty to reduce over-fitting to the data introduces bias into the regression estimates resulting in the coefficients no longer being reflective of true population-level associations with depression risk. Moreover, the purpose of prediction models is to identify the combination of predictor variables that together most accurately predict an individual’s risk of developing depression rather than considering the role of each predictor separately. Therefore, only the overall model performance statistics are provided in this paper.

## Results

After the data harmonization, there was 13.2% of the original Pelotas model’s information lost due to the unavailability of 4 of the 11 predictors in the Nepali dataset (see Table S1). All included participants had complete data on the outcome and all seven predictors included in the model (‘biological sex’, ‘caste/ethnicity’, ‘drug use’, ‘school failure’, ‘social isolation’, ‘fight involvement’, and ‘childhood maltreatment’).

The sample included in the final analysis comprised 126 Nepali adolescents. One third of the sample was female (34.1%), one-fifth was low caste (18.3%), and 43.7% were former child soldiers. A substantial proportion experienced probable childhood maltreatment (47.7%), while a smaller proportion experienced severe childhood maltreatment (27.8%). School failure was low (14.3%) among the Nepali adolescents and a smaller minority showed characteristics of being socially isolated (5.6%). No one admitted to using drugs, and behavioural problems such as getting into fights was reasonably uncommon (13.5%). The prevalence of depression at age 18 or older at wave 3 in the Nepali cohort was 19.8% (25/126 scored 14 or more on the DSRS, scores ranged from 0 to 22) comprising 18 (72%) women and 15 (60%) former child soldiers, while in the Pelotas cohort, the point prevalence of depression at age 18 was 3.1%. There was no difference in the gender, caste/ethnicity, and child soldier status between participants included and excluded from the final analysis (see Table S2).

### External validation in Nepali cohort

**Fig. 1:**
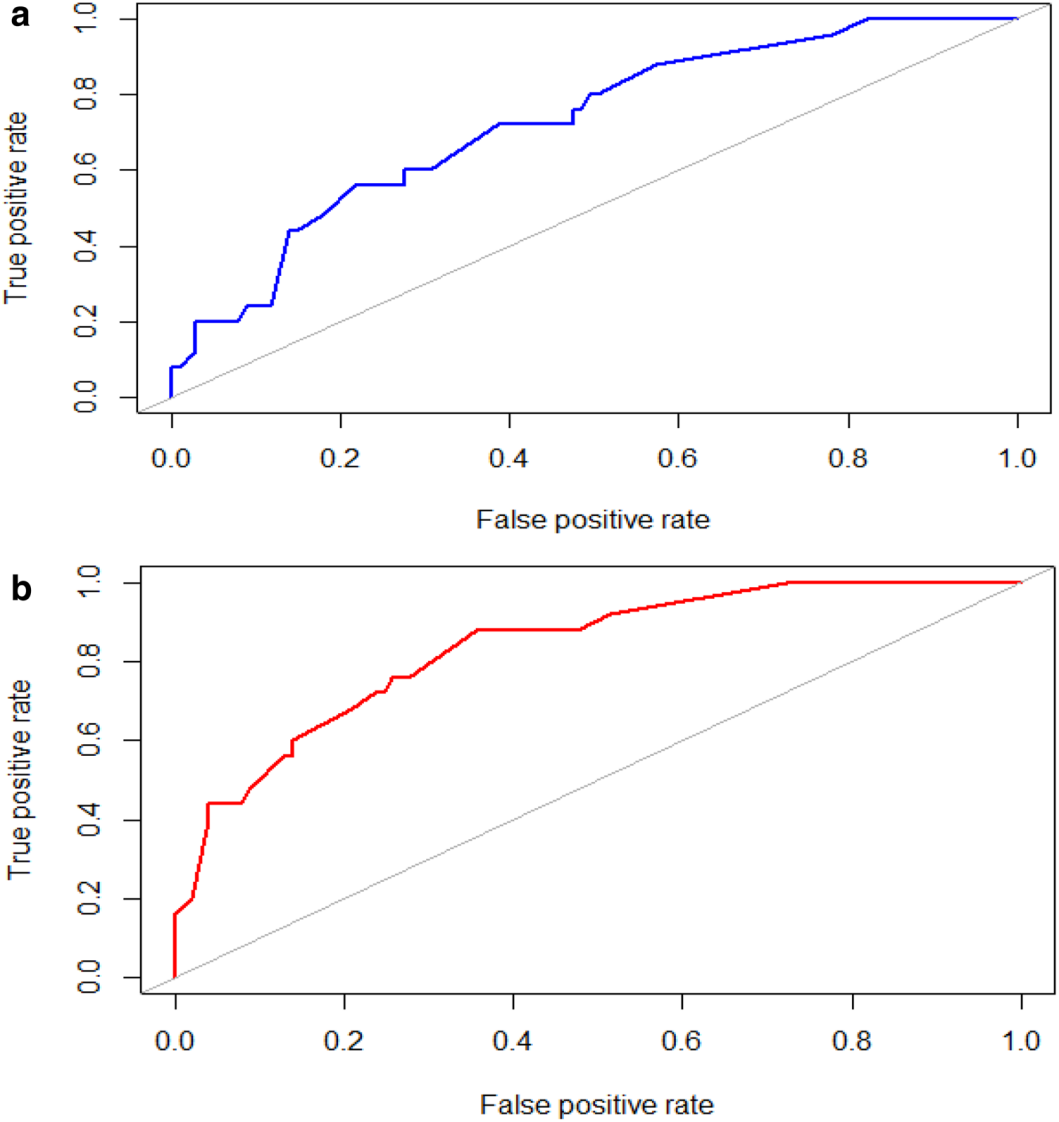
ROC curve for **a** the Pelotas model externally validated in Nepal data [area under the curve (AUC) = 0.73; bootstrap-corrected 95% confidence interval 0.62–0.83], and **b** the Pelotas model refitted in the Nepal data (AUC = 0.83; bootstrap-corrected 95% confidence interval 0.74–0.91). The *y*-axis shows the true positive rate: the proportion of adolescents correctly identified with depression. The *x*-axis shows the false positive rate: the proportion of adolescents who were wrongly identified as having depression. The grey diagonal line represents a model that discriminates the same as chance

**Fig. 2:**
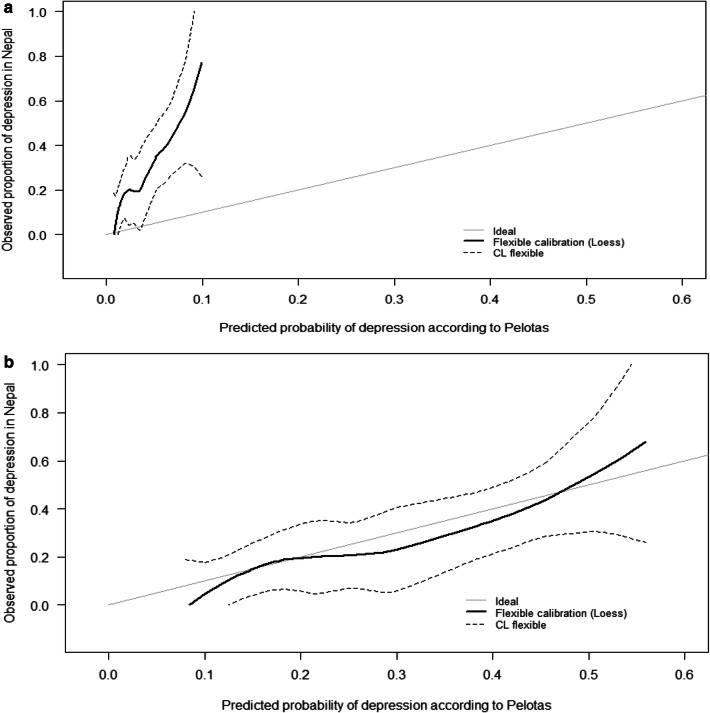
Calibration plot for **a** the Pelotas model externally validated in the Nepal dataset, and **b** when the intercept of the Pelotas model was adjusted. Graphical display of model predictions (as depicted by the black flexible calibration line with 95% confidence limits around the model predictions as dashed lines) on the *x*-axis and observed proportion of depression in the Nepali cohort on the *y*-axis. The calibration plots show how well the model predictions align with the observed rate of depression in Nepal. Perfect agreement between the predictions and the observed rate are indicated by perfect alignment on the ideal line

When applied to the Nepali cohort, the predictive model showed reasonable capacity to discriminate between individuals who developed depression in late adolescence and those who did not (AUC = 0.73; Bootstrap-corrected 95% CI 0.62–0.83; Fig. 1a). Initially, the model was not well calibrated, but this improved when the intercept was corrected (calibration-in-the-large reduced from 2.44 to 0.00) (Table 1) (Fig. 2a, b). The *p* value from the Chi-square test for unreliability of the calibration accuracy was not significant (*χ*^2^ = 0.2702, *p* = 0.874). This affirms the model achieved good calibration since there was no statistically significant difference between the model predictions and the ideal 45° line. The overall performance of the model also improved slightly after adjustment of the intercept as indicated by a reduction in the Brier score from 0.18 to 0.14 (Table 1). The final refitted model’s discriminative capacity AUC was 0.83; bootstrap-corrected 95% CI 0.74–0.91 (Fig. 1b). Its overall performance, as expected, was also better (Brier score reduced to 0.12) (Table 1).

**Table 1:** Comparison of performance metrics for the Pelotas model when externally validated in the Nepali cohort compared to its apparent and internal validation in the Pelotas cohort

Model assessment	Performance measures	Pelotas cohort		Nepali cohort		
		Apparent validation	Internal validation	Standard external validation	Adjusted external validation	Refitted model
Overall performance or model fit	Brier score	0.03	0.03	0.18	0.14	0.12
Discrimination	AUC (95% CI)	0.78 (0.73–0.82)	0.71 (*)	0.73 (0.62–0.83)	0.73 (0.62–0.83)	0.83 (0.74–0.91)
Calibration	Calibration-in-the-large	0.00	0.02	2.24	0.00	0.00
	Calibration slope	1.26	1.00	1.18	1.18	1.50

86.8% of the original Pelotas’ model’s information was available for external validation due to the availability of only 7 of the 11 predictors in the Nepali dataset

Apparent validation: the performance of the Pelotas model in the development data (in the Pelotas cohort)

Internal validation: the performance in the Pelotas cohort after controlling for over-optimism, using bootstrapping techniques

External validation (standard): the performance when applied to the Nepali sample

External validation (adjusted): the performance in the Nepali sample after the intercept was corrected

Refitted model: regression coefficients for the Pelotas model re-estimated in the Nepali dataset

AUC: area under the curve of the receiver operating characteristic (presented as a proportion). The AUC is identical to the C-statistic for binary outcomes

Brier score: quadratic scoring rule that combines calibration and discrimination—a Brier score of 0 represents a perfect model

Calibration-in-the-large reflects the model intercept. Calibration slope of a perfect model is equal to 1

*Unable to derive 95% confidence interval for the internal validation model

### Sensitivity analysis findings

There was no statistically significant interaction observed between child soldier status and the linear predictor derived from the Pelotas model in predicting depression in the Nepali cohort (*p* value = 0.802). The overall NRI result was also not significant for inclusion of child soldier status in the model (NRI = 0.0205, lower CI − 0.0829, upper CI 0.1519; standard error = 0.0603). Moreover, there was only a negligible change in the AUC value (0.01) between the Pelotas model in the Nepal cohort (AUC = 0.73) and the model with child soldier status included (AUC = 0.74). This indicates that the new model containing child soldier status as a predictor did not improve the overall Pelotas model’s predictive capacity to estimate those at higher risk of future depression. The proportion of depressed cases that moved up through the categories from low risk to high risk when the new model with child soldier status included was used was not significant (NRI+  = 0.0800, lower CI 0.0000, upper CI 0.2083; standard error = 0.0534). However, the proportion of non-depressed cases that moved down the categories from high risk to low risk was significant (NRI− = − 0.0594, lower CI − 0.1100, upper CI − 0.0098). This implies that including child soldier status in the model may be better at reducing false positives for future risk of depression in Nepal, but not false negatives.

## Discussion

### Summary of the main findings

The novel predictive model developed in an adolescent cohort in Pelotas, Brazil, comprising seven individual and social predictors (gender, caste/ethnicity, childhood maltreatment, school failure, fights, drug use, and social isolation) was able to acceptably predict clinically relevant depression in late adolescence in a Nepali adolescent cohort free of depression. The model demonstrated a good ability to differentiate adolescents who did and did not develop depression. This means that a randomly selected adolescent with depression had a higher risk prediction score than a randomly selected adolescent without depression. After adjustment, the model predictions were better aligned with the observed prevalence of depression in Nepal. There was also good overall performance despite the small sample size, and how socio-culturally divergent the Nepali adolescent population was compared to the Brazilian cohort. Moreover, the model was able to predict depression similarly for both child soldiers and war-affected civilian adolescents in Nepal inferring its ability to predict future risk of depression amongst individuals without evidence of previous depression from somewhat different backgrounds.

The Pelotas model’s ability to differentiate between adolescents who did or did not develop depression in Nepal (AUC = 0.73) was comparable to the widely used Framingham risk score which predicts whether men (AUC = 0.76) and women (AUC = 0.79) will develop cardiovascular disease [16]. It is also similar to a model developed to predict onset of major depression among adults in the US general population (AUC = 0.75) [23]. The Pelotas model has previously been assessed in two independent cohorts from high-income countries (the United Kingdom and New Zealand) with adolescents who had no evidence of previous depression [31]. The discriminative ability in the Nepali cohort was slightly better (AUC = 0.73) than its performance in the UK (AUC = 0.59) and New Zealand (AUC = 0.63) [31]; however, the 95% CI suggests the true area under the curve for the Nepali cohort can lie between 0.62 and 0.83. Nonetheless, this suggests that the Pelotas adolescent depression risk model may work better in other LMIC contexts than in high-income countries. However, further testing with suitable adjustments in a range of other LMICs is required before it can be utilized on a global scale.

### Limitations

When a model is externally validated in a different independent cohort, one of the main challenges is data harmonization. Although, we were able to closely match 7 of the 11 predictors in the Nepali dataset, differences in data collection instruments used in Nepal and Brazil resulted in imperfect harmonization. For instance, the Nepali childhood maltreatment composite variable lacked appropriate measures for ‘separation from family’, ‘feeling hated’ or ‘unwanted by close family members’ so we were not able to capture entirely the same construct. There were also differences in how depression was assessed, with a cut-off on a symptom measure (DSRS) being used to indicate clinically relevant depression in the Nepali cohort rather than a diagnosis of depression as was used in the Pelotas cohort. Nonetheless, this cut-off on the DSRS has been clinically validated in Nepal and shown to discriminate well between Nepali youth with and without a diagnosis of depression [44]. Furthermore, differences in the prevalence and reporting of risk factors may differ by context. For example, drug use was completely denied by Nepali adolescents (0% in Nepal vs 62.4% in Pelotas). In Nepal, admitting to using drugs could have reduced the chance of child soldiers being enrolled into one of the UNICEF-sponsored reintegration programs and thus tends to be underreported. Additionally, the question used to measure ‘drug use’ did not elucidate the inclusion of alcohol, marijuana, or tablets. Hence respondents probably assumed it to mean illicit drugs only, leading to non-disclosure of the use of legal substances. Moreover, we were unable to exclude individuals who had an IQ < 70 or who had not gone through puberty as was done in the original Pelotas analysis.

The Nepali adolescent cohort was not a birth cohort, hence it is unlikely to be representative of the entire Nepali adolescent population in 2007. The cohort also comprised a sample of former child soldiers of the Maoist army who voluntarily returned home after the war matched with civilian children. Hence, those who did not return home or child soldiers elsewhere may have different risks for depression [37]. The Pelotas sample was a birth cohort and risk factors were assessed if they occurred at age 15. Conversely, the cross-sectional cohort design in Nepal, meant that not all adolescents in Nepal had the same assessment age for some predictors. This along with the reasonably high loss-to-follow-up rate (43.8% at wave 3) created challenges in determining the ‘at risk’ period. Finally, the performance of the model should be interpreted with caution due to the potential for estimates to be imprecise due to the relatively small sample size.

### Implications

In Nepal, less than 1% of the Nepal government’s healthcare budget is spent on mental health and > 90% of the population in need of mental health services have no access to treatment [56]. Identification and early intervention for adolescents at higher risk of depression could potentially reduce the high suicide rates [57] and prevent morbidity [58] in this country. Using a tool comprising relatively easy-to-obtain factors to predict which adolescents are most at risk of developing depression could help target prevention initiatives.

It is also essential to conduct qualitative research with key stakeholders to gather perspectives about the feasibility and acceptability of using a depression prediction tool in Nepal. We need to advance our understanding of ways to embark upon predicting risk of depression within a culture where mental illness and experience of traumatic events are stigmatized [59]. New educational and awareness interventions that challenge social stigma towards mental health problems should thus also be considered prior to any future implementation of a risk screening tool in Nepal [60]. Moreover, careful consideration of the ethical issues surrounding the identification of adolescents at high risk of depression in low-resource settings where provision of effective interventions to prevent depression is limited is also required [61, 62].

## Conclusion

A model comprising seven demographic and social predictors developed in a middle-income country was able to reasonably predict depression among adolescents in a socio-culturally diverse, low-income country that was afflicted by humanitarian crises. We recommend testing the performance of the model in other adolescent cohorts from diverse contexts and geographical regions and with larger sample sizes before it is used in health, educational or social services’ contexts. Further exploratory research on the inclusion of more context-specific factors in the predictive model would bring added information to its replicability and generalizability across settings.

## Electronic supplementary material

Below is the link to the electronic supplementary material.

Supplementary file1 (DOCX 43 kb)
