# Supplementary material for: Predicting the risk of depression among adolescents in Nepal using a model developed in Brazil: the IDEA Project
Source: Eur Child Adolesc Psychiatry. 2020 Mar 12;30(2):213–23. doi: 10.1007/s00787-020-01505-8 (PMC7486232; doi:10.1007/s00787-020-01505-8)
Supplement: Supplementary file 1 — Supplementary file1 (DOCX 43 kb) [file 787_2020_1505_MOESM1_ESM.docx]

**SUPPLEMENTARY MATERIAL**

**Article title**: Predicting risk of depression among adolescents in Nepal using a model developed in Brazil: The IDEA Project

**Journal name**: European Child & Adolescent Psychiatry

**Author names and affiliations:**

Rachel Brathwaite^1^, Thiago Rocha^2,3^, Christian Kieling^2,3^, Kamal Gautam^4^, Suraj Koirala^4^, Valeria Mondelli^5^, Brandon Kohrt^4,6,7^, Helen L. Fisher^1,7,*^

^1^King’s College London, Social, Genetic & Developmental Psychiatry Centre, Institute of Psychiatry, Psychology & Neuroscience, London, UK.

^2^Department of Psychiatry, Universidade Federal do Rio Grande do Sul, Porto Alegre, Brazil.

^3^Child & Adolescent Psychiatry Division, Hospital de Clínicas de Porto Alegre, Brazil.

^4^Transcultural Psychosocial Organization Nepal (TPO Nepal), Nepal.

^5^King’s College London, Department of Psychological Medicine, Institute of Psychiatry, Psychology & Neuroscience, London, UK.

^6^Division of Global Mental Health, George Washington University, Washington DC., USA.

^7^These authors contributed equally to this work

* Correspondence to: Dr Helen L. Fisher, SGDP Centre, IoPPN, 16 De Crespigny Park, London, SE5 8AF, UK. Tel: +44(0)2078485430. Fax: +44(0)2078480866. Email: helen.2.fisher@kcl.ac.uk

**Table S1**. **Description of predictors and depression outcome measures in the Pelotas cohort and matching variables in the Nepali cohort.**

| **No.** | **Pelotas cohort predictors and description** | **Categories** | **Matching variable available in Nepali cohort?** | **Nepali cohort matching predictor variable and description**  **[data collection wave from child soldiers longitudinal research, assessment tool used]** | **Categories** |
| --- | --- | --- | --- | --- | --- |
| **1** | **Biological sex** | ***Male*** | Yes | **Biological sex**  **[wave 1, demographics]** | ***Male*** |
|  |  | Female |  |  | Female |
| **2** | **Skin colour**  Self-assigned skin colour | ***White*** | Indirectly | **Ethnicity/caste**  **[wave 1, demographics]** | ***Janajati and High Caste*** |
|  |  | Non-white |  |  | Dalit  (this is the historically marginalised at risk group in Nepal) |
| **3** | **Childhood maltreatment^a^**  Responses to seven dichotomous questions on lifetime exposure to emotional, physical, or sexual abuse and child neglect at age 15 years old:   1. Have you ever been separated from your parents to be cared for by someone else? 2. Have you ever had, in your home, fights with physical aggression between adults or an adult who assaulted a child or teenager? 3. Have you ever not had enough food at home or had to wear dirty or torn clothes because you had no others? 4. Have you ever thought or felt that your parents wished you were never born? 5. Have you ever thought or felt that someone in your family hated you? 6. Did an adult in your family or someone who was taking care of you ever beat you in a way that has hurt you or left you with marks? 7. Did someone ever try to touch you in a sexual way, or tried to make you touch them against your will, threatening you or hurting you? | ***None***  *(no positive responses)* | Partially | **Childhood maltreatment**  Responses to 4 dichotomous questions regarding lifetime exposure to domestic violence, physical and sexual abuse and/or neglect before age 18 years:   1. ‘Ever experience of parents fighting with each other’ **[wave 1, Kiddie SADS-traumatic events inventory]^b^** 2. ‘Do you have access to food’ and ‘Do you have access to clothing’ **[wave 2, personal and household information]^c^** 3. ‘Ever experienced physical abuse by parents.’ **[wave 1, Kiddie SADS -traumatic events inventory]^b^** 4. ‘Lifetime experience of sexual violence’ **[wave 2, Kiddie SADS-traumatic events inventory]^b^** | ***None***  *(no positive responses)* |
|  |  | Probable  (1 positive response) |  |  | Probable  (1 positive response) |
|  |  | Severe  (2 or more positive responses) |  |  | Severe  (2 or more positive responses) |
| **4** | **School failure** | ***No school failure***  *(Child progressed to next level if achieved predetermined score at end of school year).* | Indirectly | **School failure**  **[information from all waves, demographics]** | ***No school failure***  *(Currently in school or passed the School Leaving Certificate (SLC))* |
|  |  | Failing at school  (Child was retained in school to repeat the same school year if failed to achieve a predetermined score at end of school year). |  |  | School failure  (Not currently in school and did not pass the SLC) |
| **5** | **Social isolation** | ***No***  (Normally meets up with friends to chat, play or do other things) | Indirectly | **Social isolation**  **[wave 1, daily functioning]^d^** | ***No***  *(Never or a little difficult to spend time with friends (return home with friends, to talk with friends)* *in the past 2 weeks)* |
|  |  | Yes  *(Does not normally meets up with friends to chat, play or do other things)* |  |  | Yes  (Sometimes or usually difficult to spend time with friends (return home with friends, to talk with friends) in the past 2 weeks) |
| **6** | **Fights** | ***No***  *(Never got into a physical fight where someone got hurt in the last year)* | Yes | **Fights**  **[wave 1, Kiddie SADS-traumatic events inventory]^b^** | ***No***  *(Experience of beating a person: None or witnessed)* |
|  |  | Yes  (Got into a physical fight where someone got hurt in the last year) |  |  | Yes  (Experience of beating a person: Suffered or committed) |
| **7** | **Ran away** | ***No***  *(Never ran away from home)* | No | n/a | n/a |
|  |  | Yes  (Ran away from home) |  |  | n/a |
| **8** | **Drug use**  Responses to dichotomous questions about lifetime use of alcohol, tobacco, cannabis, cocaine and inhalants. | ***No***  *(Otherwise)* | Indirectly | **Drug abuse**  Drug abuse in entire lifetime  **[wave 2, Kiddie SADS-traumatic events inventory]^b^** | ***No***  *(None or witnessed drug abuse)* |
|  |  | Yes  (any positive answer) |  |  | Yes  (Personally committed drug abuse) |
| **9** | **Relationship with mother**  Responses to question on ‘How do you rate your relationship with your mother’. | ***Great*** | No | n/a | n/a |
|  |  | Very good, Good, Regular, or Bad |  |  | n/a |
| **10** | **Relationship with father**  Responses to question on ‘How do you rate your relationship with your father’. | ***Great*** | No | n/a | n/a |
|  |  | Very good, Good, Regular, or Bad |  |  | n/a |
| **11** | **Relationship between parents**  Responses to question on ‘How do you rate the relationship between your mother and father’. | ***Great*** | No | n/a | n/a |
|  |  | Very good, Good, Regular, or Bad |  |  | n/a |
| TOTAL | 11/11 |  | 7/11 |  |  |
| **OUTCOME** | |  |  |  |  |
|  | **Depression diagnosis at age 18**  Evaluation of depressive episode diagnosis with DSM-IV-TR criteria in the previous two weeks^e^ | ***No*** | Yes | **Depression diagnosis at age 18 or older**  Evaluation of depressive symptoms in the past week, using the Nepali-validated DSRS.  **[wave 3, Depression Self-Rating Scale]^f^** | ***No***  *(DSRS total score <14)* |
|  |  | Yes |  |  | Yes  (DSRS total score ≥14) |

***Reference category of each variable are in bold italics***. ^a^Questions on childhood maltreatment asked in the Pelotas 15 years’ assessment [1]. ^b^Kiddie SADS-Kiddie Schedule for Affective Disorders and Schizophrenia Present and Lifetime Version [2]; n/a - no matching variable available in dataset. ^c^Alternative data collection approach for socioeconomic status in settings with predominantly informal economy and subsistence labour [3]. ^d^Tool developed based on the experiences of child soldiers returning to their communities and experiences of civilian children after the war [4]. ^e^ In Pelotas, trained psychologists diagnosed adolescents using a tool created from the MINI International Neuropsychiatric Interview [5]. ^f^Nepali-validated version of the DSRS - Depression Self-Rating Scale [6, 7]. SLC - School Leaving Certificate. DSM-IV-TR - Diagnostic and Statistical Manual of Mental Disorders-Fourth Edition (Text Revision).

**Table S2. Comparison of characteristics of participants included and excluded from the final analysis.**

| **Characteristic** | **Categories** | **Included in final analysis**  **(N=126)** | **Excluded from final analysis**  **(N=390)** | **χ^2^ (p value)** |
| --- | --- | --- | --- | --- |
|  |  | **n (%)** | **n (%)** |  |
| **Gender** | Male | 83 (65.9) | 236 (60.5) | 1.1593 (0.282) |
|  | Female | 43 (34.1) | 154 (39.5) |  |
| **Ethnicity** | High Caste or Janajati | 103 (81.8) | 288 (73.9) | 3.2377 (0.072) |
|  | Dalit | 23 (18.3) | 102 (26.2) |  |
| **Soldier status** | Child Soldier | 55 (43.7) | 203 (52.1) | 2.6882 (0.101) |
|  | Child Civilian | 71 (56.4) | 71 (56.4) |  |

χ^2^ Pearson chi-squared test comparing difference between included and excluded participants by key characteristics.

Total sample at wave 1 (baseline)

**N=516**

EXCLUDED:

- No depression assessment at wave 2 **(n=60)**
- No depression assessment at wave 3 **(n=184)**

Total sample size remaining after loss to follow-up at waves 2 and 3: **N=272**

EXCLUDED:

- Younger than 18 at wave 3 **(n=19)**
- 18 or older at wave 2 **(n=43)**

Total sample size remaining after exclusions based on age: **N=210**

EXCLUDED:

- Above cut-off for depression at wave 2 **(n=37)**
- Above cut-off for depression at wave 1 **(n=47)**

Total sample size included in final analysis:

**N=126**

(71 controls + 55 soldiers)

**Fig. S1 Flow chart showing selection of the final sample of the Nepali adolescent cohort included in the analysis.**

**REFERENCES**

1. Rocha TB-M, Hutz MH, Salatino-Oliveira A, Genro JP, Polanczyk GV, Sato JR, Wehrmeister FC, Barros FC, Menezes AMB, Rohde LA, Anselmi L, Kieling C (2015) Gene-environment interaction in youth depression: Replication of the 5-HTTLPR moderation in a diverse setting. Am J Psychiatry 172:978-985. <https://doi.org/10.1176/appi.ajp.2015.14070896>
2. Kaufman J, Chambers W, Puig-Antich J, Birmaher B, Rao U, Ryan ND (1996) Kiddie-SADS-Present and Lifetime Version. Pittsburgh, PA: University of Pittsburgh.
3. Kohrt BA, Jordans MJ, Tol WA, Speckman RA, Maharjan SM, Worthman CM, Komproe IH (2008) Comparison of mental health between former child soldiers and children never conscripted by armed groups in Nepal. JAMA 300:691-702. <https://doi.org/10.1001/jama.300.6.691>
4. Kohrt BA, Jordans MJD, Tol WA, Perera E, Karki R, Koirala S, Upadhaya N (2010) Social ecology of child soldiers: child, family, and community determinants of mental health, psychosocial well-being, and reintegration in Nepal. Transcult Psychiatry 47:727-753. https://doi.org/10.1177/1363461510381290
5. Amorim P (2000) Mini International Neuropsychiatric Interview (MINI): validação de entrevista breve para diagnóstico de transtornos mentais. Rev Bras Psiquiatr 22:106-115. https://doi.org/10.1590/S1516-44462000000300003
6. Birleson P (1981) The validity of depressive disorder in childhood and the development of a self-rating scale: a research report. J Child Psychol Psychiatry 22:73-88. https://doi.org/10.1111/j.1469-7610.1981.tb00533.x
7. Kohrt BA, Jordans MJD, Tol WA, Luitel NP, Maharjan SM, Upadhaya N (2011) Validation of cross-cultural child mental health and psychosocial research instruments: adapting the Depression Self-Rating Scale and Child PTSD Symptom Scale in Nepal. BMC Psychiatry 11:127. https://doi.org/10.1186/1471-244X-11-127
